# CT-based nomogram for estimating visceral pleural invasion in solid pulmonary adenocarcinoma nodules with pleural contact: a retrospective single-center study

**DOI:** 10.1186/s12880-026-02458-1

**Published:** 2026-05-26

**Authors:** Rui Xu, Jianing Tang, Qianyao Yuan, Mengjiao Ding, Jingjing Zhang, Wenjun Yao, Dai Zhang, Hong Zhao

**Affiliations:** Department of Radiology, The Second Affiliated Hospital of Anhui Medical University, Anhui, Hefei 230601 China

**Keywords:** Visceral pleural invasion (VPI), Lung adenocarcinoma, Computed tomography (CT), Nomogram

## Abstract

**Background:**

Visceral pleural invasion (VPI) is an adverse prognostic factor in lung adenocarcinoma. Accurate preoperative estimation of VPI risk in solid tumors with pleural contact may provide useful supplementary information during preoperative assessment.

**Methods:**

This was a retrospective study of 162 patients. All patients had surgically resected, pathologically confirmed solid lung adenocarcinoma in pleural contact between November 2018 and August 2025. Patients were classified into VPI-positive and VPI-negative groups based on postoperative pathology. The patients were randomly divided into a training cohort and a testing cohort at a ratio of 7:3 for internal validation. Multivariable logistic regression was performed to identify independent risk factors for VPI. A nomogram prediction model was developed based on the multivariable analysis. Its predictive performance was then evaluated using receiver operating characteristic (ROC) curves, calibration curves, and decision curve analysis (DCA).

**Results:**

Of the 162 patients with solid lung adenocarcinoma nodules, 58 had VPI confirmed by pathology. Multivariate logistic regression analysis identified spiculation, pleural indentation, and pleural contact length as independent risk factors for predicting VPI. A nomogram based on these three CT features showed good discriminative performance in both the training and testing cohorts, with AUCs of 0.901 (95% CI: 0.846–0.956) and 0.864 (95% CI: 0.737–0.937), respectively. Calibration curves showed the predictions matched observations well. Decision curve analysis suggested potential utility in preoperative risk assessment.

**Conclusion:**

Among patients with solid lung adenocarcinoma nodules with pleural contact, we developed a nomogram based on routinely assessed CT semantic features to estimate the preoperative probability of VPI. The model is intended as a practical supplementary tool for preoperative VPI risk estimation.

**Supplementary Information:**

The online version contains supplementary material available at 10.1186/s12880-026-02458-1.

## Introduction

Lung cancer is a leading cause of global cancer incidence and mortality [1, 2]. Its rates continue to rise, presenting a serious public health threat. Lung adenocarcinoma (LUAD) is the most common histological subtype of non-small cell lung cancer (NSCLC) [3]. Although treatment strategies have advanced in recent years, the overall prognosis for lung cancer remains poor. Recent population-based data indicate that the overall 5-year relative survival for lung cancer is approximately 22%, with substantial variation according to stage at diagnosis [1].

Visceral pleural invasion (VPI) is an important adverse prognostic factor in NSCLC [4–6]. It directly impacts tumor staging, treatment decisions, and outcomes. According to the 9th edition of the tumor–node–metastasis (TNM) staging system for lung cancer, when VPI is present, tumors measuring ≤ 3 cm are upstaged from T1 to T2a. This results in a shift of the clinical stage from IA to IB [7]. In current clinical practice, the extent of resection for small-sized peripheral NSCLC is increasingly informed by contemporary randomized evidence. Recent trials, including JCOG0802/WJOG4607L and CALGB/Alliance 140,503, have supported sublobar resection in selected patients, although the role of VPI in determining the adequacy of limited resection remains controversial [8, 9]. Jiang et al. [10] showed that the presence of VPI significantly worsens prognosis in patients with tumors ≤ 3 cm in diameter. Similarly, Hung et al. [11] reported that visceral pleural invasion at the PL2 level predicts higher recurrence and lower survival in NSCLC.

Chest computed tomography (CT) plays a key role in the preoperative assessment of lung adenocarcinoma [12, 13]. Previous studies have shown that several CT features—such as tumor location and size, lobulation, and pleural indentation—are associated with VPI [14, 15]. Previous CT-based studies on preoperative VPI prediction have largely been conducted in broader mixed cohorts including subsolid and solid nodules. Therefore, their findings may not be directly generalizable to solid pulmonary adenocarcinoma nodules with pleural contact, a clinically specific subgroup characterized by direct tumor–pleura apposition and potentially distinct VPI risk patterns. Compared with subsolid nodules, solid lung adenocarcinomas generally exhibit greater invasiveness and are more likely to develop VPI [18]. In addition, pleural contact length has been reported as an independent predictor of VPI [17, 19], potentially because direct tumor–pleura apposition reduces the buffering effect of intervening normal lung parenchyma and may facilitate pleural invasion.

At present, dedicated prediction models for solid lung adenocarcinoma nodules with pleural contact are still uncommon, even though this subgroup is clinically relevant and may carry a higher risk of pleural invasion. When a solid tumor abuts the pleura, the tumor–pleura interface is different from that seen in subsolid or mixed cohorts, and local extension becomes a more realistic concern; as a result, models developed in those broader populations may not translate well to pleura-abutting solid nodules. Therefore, the aim of the present study was to develop and internally validate a CT-based nomogram for preoperative estimation of VPI risk in solid pulmonary adenocarcinoma nodules with pleural contact using routinely assessed semantic CT features.

## Materials and methods

### Patients

**Fig. 1:**
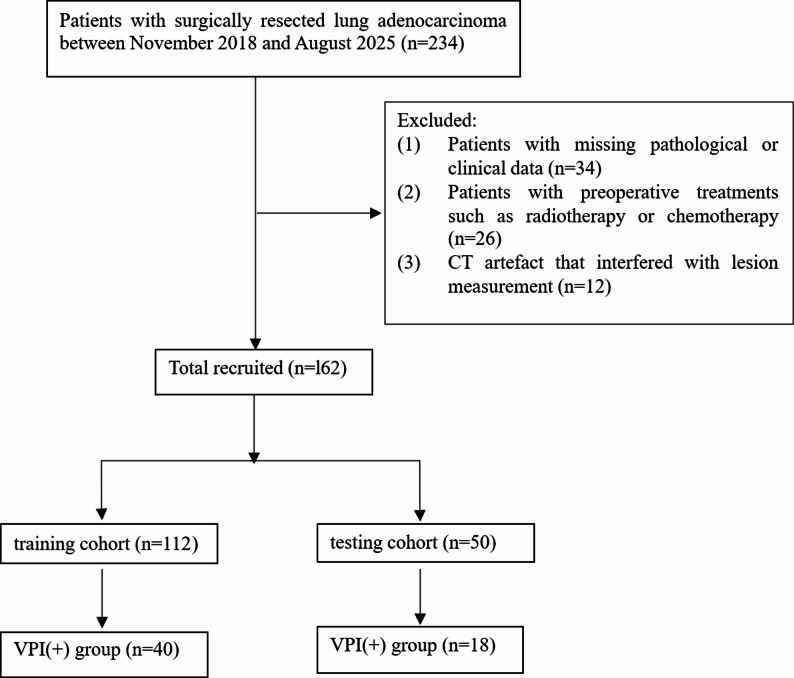
The inclusion/exclusion flow diagram. Abbreviations: CT = computed tomography. VPI = visceral pleural invasion

This retrospective study was approved by the Institutional Review Board of the hospital, and the requirement for written informed consent was waived. For model development and internal validation, the included patients were randomly divided into a training cohort and a testing cohort at a ratio of 7:3 within the same institutional dataset. The study retrospectively analyzed the CT imaging features, clinical, and pathological data of primary pulmonary adenocarcinoma patients confirmed by surgical pathology at the hospital from November 2018 to August 2025. The inclusion criteria were as follows: (1) Pathologically confirmed diagnosis of primary pulmonary adenocarcinoma. (2) Maximum tumor diameter ≤ 3 cm. (3) Complete chest CT scan data within 1 month prior to surgery. (4) Chest CT imaging showing solid nodules with pleural contact. The exclusion criteria were as follows: (1) Patients with missing pathological or clinical data. (2) Patients with preoperative treatments such as radiotherapy or chemotherapy. (3) CT artefacts that interfered with lesion measurement. The inclusion/exclusion flow diagram is shown in Fig. 1. Based on pathological findings, patients were classified into VPI-positive and VPI-negative groups. Clinicopathological and imaging data were collected for further analysis. At our institution, the choice between lobectomy and sublobar resection was made according to routine multidisciplinary clinical judgment, taking into account tumor size and location, radiologic invasiveness, the anticipated ability to achieve an adequate surgical margin, pulmonary reserve, and patient comorbidities.

### Pathological evaluation

All surgically resected specimens underwent routine pathological examination with hematoxylin and eosin (H&E) staining. The tumor–pleura interface on the resected specimen was routinely assessed by pathologists to determine the extent of pleural involvement. When the relationship between tumor cells and the visceral pleural elastic layer could not be confidently determined on H&E sections, additional elastic fiber staining was performed. According to the 9th edition of the TNM classification for lung cancer, PL0 was defined as no tumor invasion beyond the visceral pleural elastic layer, PL1 as tumor invasion beyond the elastic layer, and PL2 as tumor extension to the visceral pleural surface. Cases with PL1 or PL2 were classified as VPI-positive, whereas PL0 cases were classified as VPI-negative. Tumor invasion of the parietal pleura (PL3) was not included in the present study.

### Imaging technique

All CT scans were performed using a SOMATOM Force scanner (Siemens Healthineers, Forchheim, Germany) during end-inspiratory breath-hold. The scanning parameters were as follows: tube voltage, 120 kV; tube current, 150–200 mA; collimation/slice thickness, 5 mm; and slice interval, 5 mm. Raw data were acquired with 5-mm collimation and additionally reconstructed into 1.25-mm thin sections (interval, 1.25 mm) for lung-window analysis. The scanning range extended from the thoracic inlet to the level of the diaphragm. After scanning, the raw images were transferred to a post-processing system. CT images were reconstructed with the following window settings: lung window (window width, 1200 HU; window level, − 600 HU) and mediastinal window (window width, 400 HU; window level, 40 HU).

### Image analysis

**Fig. 2:**
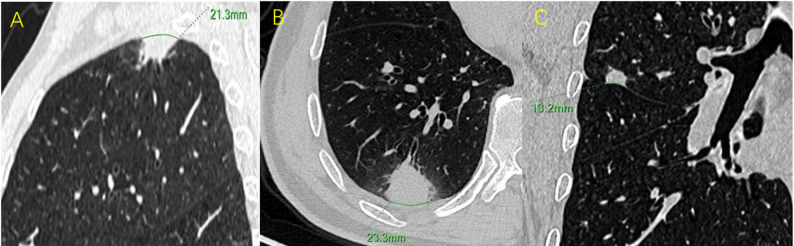
(**a–c**) Sagittal, axial, and coronal CT images of solid pulmonary adenocarcinoma nodules with pleural contact pathologically confirmed as VPI-positive; preoperative CT shows pleural contact lengths of 21.3 mm, 23.3 mm, and 13.2 mm. Abbreviations: VPI, visceral pleural invasion; CT, computed tomography

CT images were independently reviewed by two senior radiologists, each with more than 10 years of clinical experience. Both radiologists were blinded to the patients’ pathological results. In cases of disagreement, a consensus was reached through discussion. For nodule type classification, a solid nodule was defined as a nodule in which the solid component accounted for > 80% of the entire nodule [20]. The evaluated CT semantic features were predefined based on prior literature on CT predictors of visceral pleural invasion and tumor–pleura interaction, as well as their routine availability and interpretability in clinical practice. The following CT features were evaluated: (1) nodule location (right upper/middle/lower lobe; left upper/lower lobe); (2) nodule size (the mean of the maximum long-axis diameter and the perpendicular short-axis diameter on the largest axial slice); (3) lobulation; (4) spiculation; (5) air bronchogram; (6) vacuole sign (air-containing area < 5 mm); (7) pleural indentation; (8) cavity (air-containing area ≥ 5 mm); (9) calcification; (10) vascular convergence; (11) pleural contact length. The pleural contact length was defined as the longest continuous curvilinear interface between the nodule and the visceral pleura. It was measured on lung-window images using multiplanar reconstruction (axial, coronal, and sagittal planes) on the Philips IntelliSpace Portal workstation (v6.0.5.0299), with the maximum interface length manually traced on the three-dimensional reconstructed images with MPR assistance [17, 21] (Fig. 2).

### Statistical analysis

Statistical analysis was performed using SPSS version 30.0 and R software (version 4.4.1). The Kolmogorov–Smirnov test and the Shapiro–Wilk test were used to assess the normality of quantitative data. Data with a normal distribution were expressed as mean ± standard deviation (x ± s), and comparisons between groups were performed using the independent-sample t-test. Non-normally distributed data were expressed as median and interquartile range [M (P25, P75)], and comparisons between groups were performed using the Mann-Whitney U test. Categorical data were expressed as counts and percentages (%), and comparisons between groups were conducted using the Chi-square test. Variables showing statistically significant differences in univariate analysis were subsequently entered into the multivariable logistic regression model to identify independent predictors of VPI. To improve methodological transparency, we also evaluated model stability by considering the events-per-variable (EPV) ratio and assessed collinearity among candidate predictors using the variance inflation factor (VIF), with VIF ≤ 5 indicating no problematic multicollinearity in the final model. A nomogram model for predicting visceral pleural invasion (VPI) in lung adenocarcinoma was constructed using the “rms” package in R software. Calibration curves were applied to assess the agreement between the predicted probability of VPI and the observed incidence. In the training cohort, bootstrap resampling (1,000 repetitions) was used to obtain optimism-corrected (bias-corrected) calibration. In the testing cohort, calibration curves were estimated with bootstrap resampling (1,000 repetitions) to reduce sampling variability. Clinical decision curve analysis (DCA) was performed to evaluate the clinical utility of the nomogram.

## Results

### Clinical characteristics and imaging features of patients

A total of 162 patients were included in this study, comprising 58 patients in the VPI-positive group and 104 patients in the VPI-negative group, with an overall VPI incidence of 35.8%. Among all 162 patients, 122 tumors (75.3%) measured ≤ 2 cm and 40 tumors (24.7%) measured > 2–3 cm; in the training cohort, 83 of 112 tumors (74.1%) were ≤ 2 cm and 29 (25.9%) were > 2–3 cm, whereas in the testing cohort, 39 of 50 tumors (78.0%) were ≤ 2 cm and 11 (22.0%) were > 2–3 cm. For internal validation, the cohort was randomly divided into a training cohort (70%, *n* = 112) and a testing cohort (30%, *n* = 50). In the training cohort, the VPI-positive group comprised 23 males and 17 females with a mean age of 62.4 ± 8.8 years, whereas the VPI-negative group included 32 males and 40 females with a mean age of 59.7 ± 11.7 years. No statistically significant differences were observed between the two groups in clinical variables, including sex, age, history of surgery, smoking history, and surgical procedure (all *P* > 0.05). Regarding CT imaging features, significant differences were identified between the VPI-positive and VPI-negative groups in nodule size, spiculation, pleural indentation, and pleural contact length (all *P* < 0.05). Compared with the VPI-negative group, patients with VPI-positive solid lung adenocarcinoma nodules had significantly larger tumor sizes (18.9 ± 4.7 mm vs. 14.5 ± 4.9 mm, *P* < 0.001). In addition, spiculation (70.0% vs. 26.4%, *P* < 0.001) and pleural indentation (55.0% vs. 34.7%, *P* = 0.037) were more frequently observed in the VPI-positive group. The pleural contact length was also significantly greater in the VPI-positive group [19.1 (14.4, 22.7) mm vs. 9.2 (5.6, 13.6) mm, *P* < 0.001]. By contrast, no significant differences were found in other CT features, including nodule location, lobulation, air bronchogram, vacuole sign, cavity, calcification, emphysema, or vascular convergence (all *P* > 0.05), as summarized in Tables 1 and 2.

**Table 1:** Clinical characteristics of patients in training and testing cohorts

parameters	Training cohort			Testing cohort		
	VPI(+) *n* = 40	VPI(-) *n* = 72	*P*	VPI(+) *n* = 18	VPI(-) *n* = 32	*P*
Gender			0.185			0.264
Male	23(57.5)	32(44.4)		13(72.2)	18(56.2)	
Female	17(42.5)	40(55.6)		5(27.8)	14(43.8)	
Age(year)	62.4 ± 8.8	59.7 ± 11.7	0.212	59.3 ± 8.9	59.7 ± 8.7	0.865
Operation history			0.75			0.368
Absent	30(75)	52(72.2)		14(77.8)	21(65.6)	
Present	10(25)	20(27.8)		4(22.2)	11(34.4)	
Smoking history			0.9			0.05
Absent	38(95)	68(94.4)		14(77.8)	31(96.9)	
Present	2(5)	4(5.6)		4(22.2)	1(3.1)	
Type of surgery			0.077			0.375
Sublobar resection	4(10)	17(23.6)		6(33.3)	7(21.9)	
Lobectomy	36(90)	55(76.4)		12(66.7)	25(78.1)	

**Table 2:** CT imaging features of patients in training and testing cohorts

parameters	Training cohort			Testing cohort		
	VPI(+) *n* = 40	VPI(-) *n* = 72	*P*	VPI(+) *n* = 18	VPI(-) *n* = 32	*P*
Nodule location			0.939			0.574
Right lower lobe	9(22.5)	17(23.6)		3(16.7)	7(21.9)	
Right middle lobe	7(17.5)	12(16.7)		1(5.6)	3(9.4)	
Right upper lobe	10(25)	17(23.6)		5(27.8)	9(28.1)	
Left lower lobe	9(22.5)	13(18.1)		2(11.1)	7(21.9)	
Left upper lobe	5(12.5)	13(18.1)		7(38.9)	6(18.8)	
Nodule size(mm)	18.9 ± 4.7	14.5 ± 4.9	< 0.001	15.3 ± 4.9	17 ± 4.8	0.232
Spiculation			< 0.001			0.001
Absent	12(30)	53(73.6)		5(27.8)	24(75)	
Present	28(70)	19(26.4)		13(72.2)	8(25)	
Lobulation			0.629			0.768
Absent	18(45)	29(40.3)		6(33.3)	12(37.5)	
Present	22(55)	43(59.7)		12(66.7)	20(62.5)	
Air bronchogram			0.734			0.977
Absent	29(72.5)	50(69.4)		14(77.8)	25(78.1)	
Present	11(27.5)	22(30.6)		4(22.2)	7(21.9)	
Vacuole sign			0.069			0.684
Absent	34(85)	50(69.4)		15(83.3)	28(87.5)	
Present	6(15)	22(30.6)		3(16.7)	4(12.5)	
Pleural indentation			0.037			0.022
Absent	18(45)	47(65.3)		7(38.9)	23(71.9)	
Present	22(55)	25(34.7)		11(61.1)	9(28.1)	
Cavity			0.616			0.53
Absent	38(95)	70(97.2)		18(100)	30(93.8)	
Present	2(5)	2(2.8)		0	2(6.3)	
Calcification			1			1
Absent	39(97.5)	71(98.6)		17(94.4)	31(96.9)	
Present	1(2.5)	1(1.4)		1(5.6)	1(3.1)	
Emphysema			0.491			0.659
Absent	31(77.5)	53(73.6)		15(83.3)	25(78.1)	
Present	9(22.5)	19(26.4)		3(16.7)	7(21.9)	
Vascular convergence			0.225			0.83
Absent	29(72.5)	44(61.1)		5(27.8)	8(25)	
Present	11(27.5)	28(38.9)		13(72.2)	24(75)	
Pleural contact length (mm)	19.1 (14.4,22.7)	9.2 (5.6,13.6)	< 0.001	16.3 ± 7.3	11.9 ± 6.1	0.026

VPI, visceral pleural invasion; CT, computed tomography

### Interobserver agreement of CT features

The measurements of CT imaging features by two observers for the dataset showed excellent consistency for quantitative parameters (ICC ranging from 0.991 to 0.994) and strong consistency for qualitative indicators (Kappa values ranging from 0.873 to 1.000) (Supplementary Table 1).

### Multivariate logistic regression analysis and nomogram construction

**Fig. 3:**
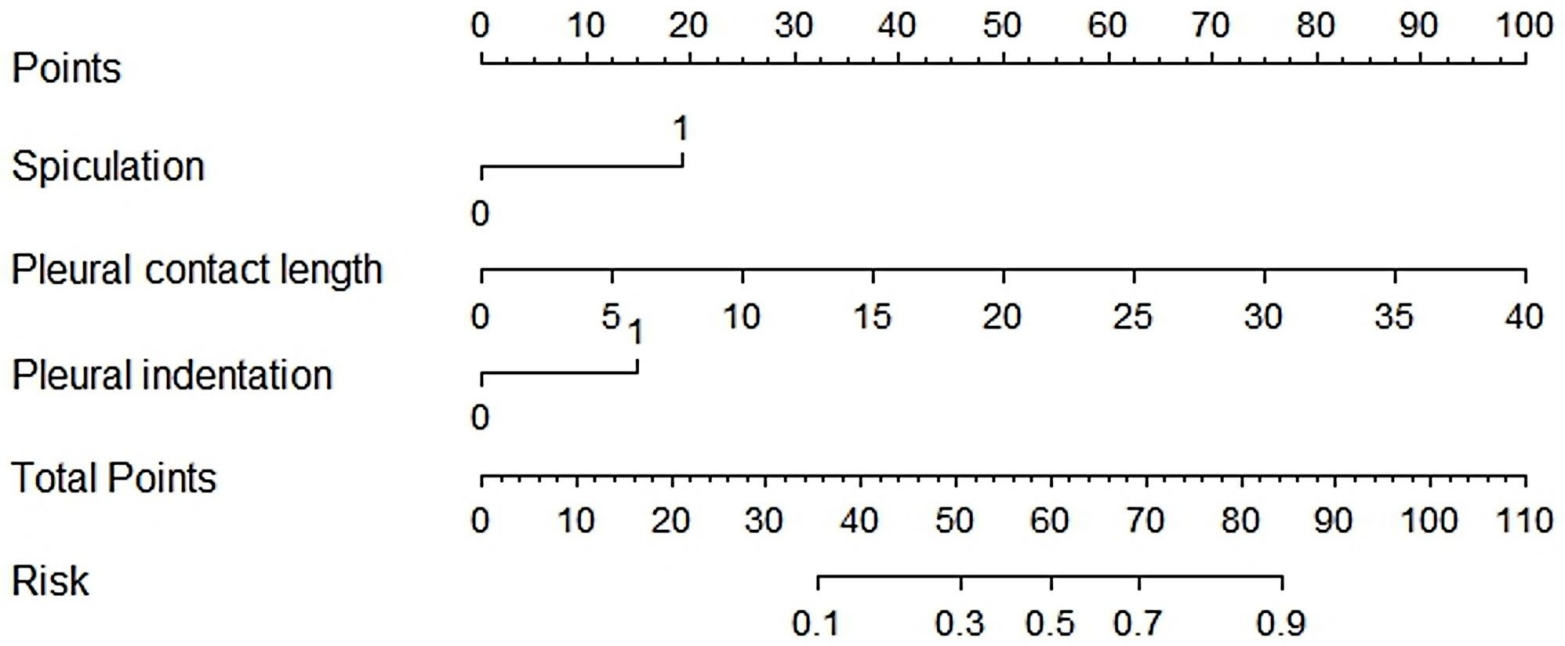
Nomogram for prediction of VPI based on training cohort. Abbreviations: VPI= visceral pleural invasion

Features showing significant differences in univariate analysis (*P* < 0.05) were entered into the multivariate logistic regression model. Spiculation (odds ratio [OR] = 5.593, 95% confidence interval [CI]: 1.815–17.239), pleural indentation (OR = 3.770, 95% CI: 1.188–11.970), and pleural contact length (OR = 1.247, 95% CI: 1.120–1.387) remained independently associated with VPI in solid lung adenocarcinoma nodules with pleural contact (Table 3). A nomogram incorporating these predictors was subsequently developed based on the fitted regression coefficients to provide an individualized estimate of VPI risk (Fig. 3). The nomogram assigns points to each predictor according to its regression coefficient, such that variables with larger effects contribute proportionally more to the overall risk estimate. For an individual patient, the value of each CT feature is mapped to the corresponding points on the nomogram scale, and the points are summed to yield a total score. This total score is then translated into the estimated probability of VPI, enabling individualized preoperative risk prediction.

**Table 3:** Logistic regression models showing variables independently associated with VPI in training cohort

Variables	OR	95%CI	*P*
Spiculation	5.593	1.815–17.239	0.003
Pleural indentation	3.77	1.188–11.97	0.024
Pleural contact length	1.247	1.12–1.387	< 0.001

OR, odds ratio; CI, Confidence interval

### Evaluation of model performance

**Fig. 4:**
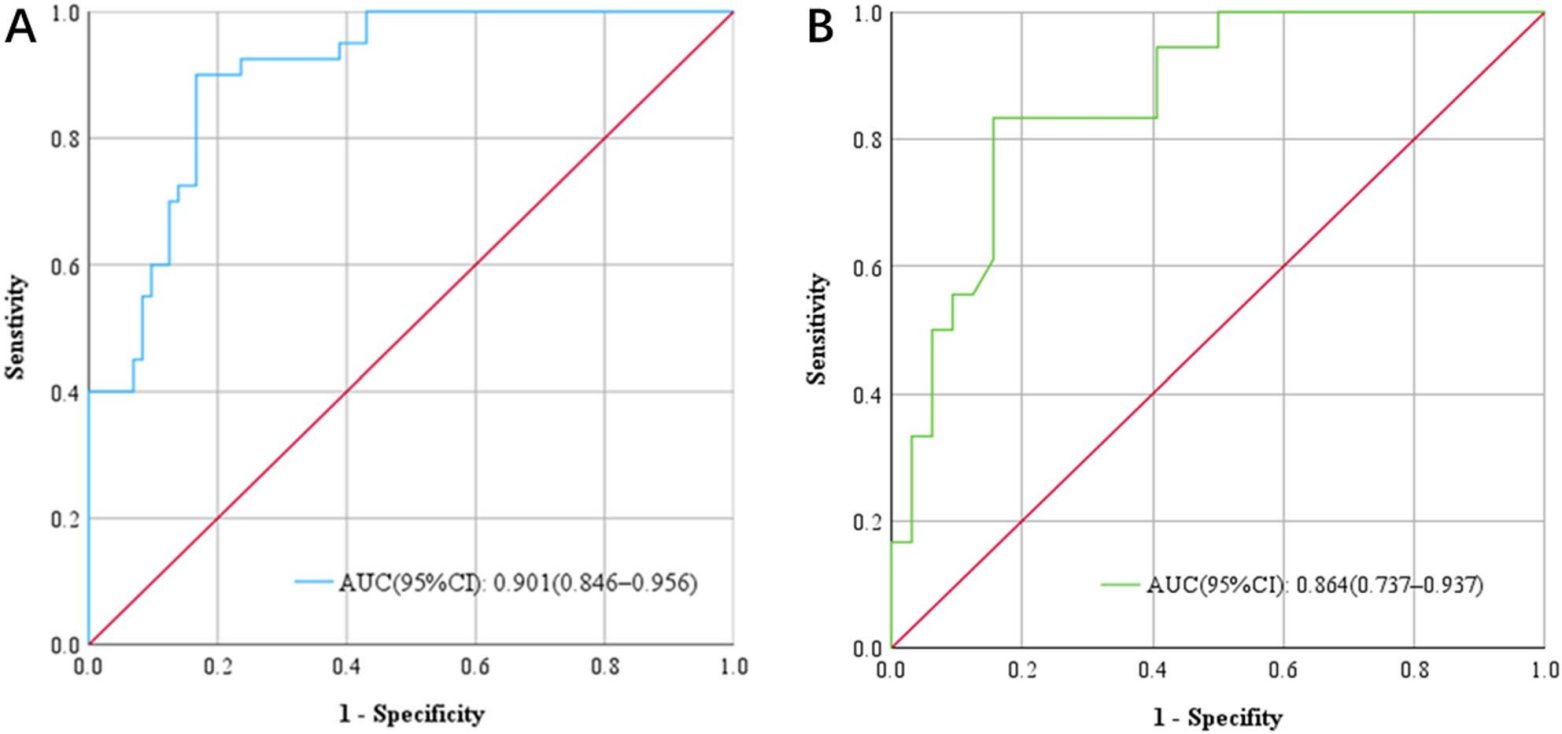
ROC curve and AUC of the predictive model. For training cohort (**A**), area under the curve (AUC) = 0.901 with 95% CI 0.846–0.956. For testing cohort (**B**), AUC = 0.864 with 95% CI 0.737–0.937. Abbreviations: ROC =receiver operating characteristic; VPI= visceral pleural invasion; AUC =area under the curve; CI =confidence interval

**Fig. 5:**
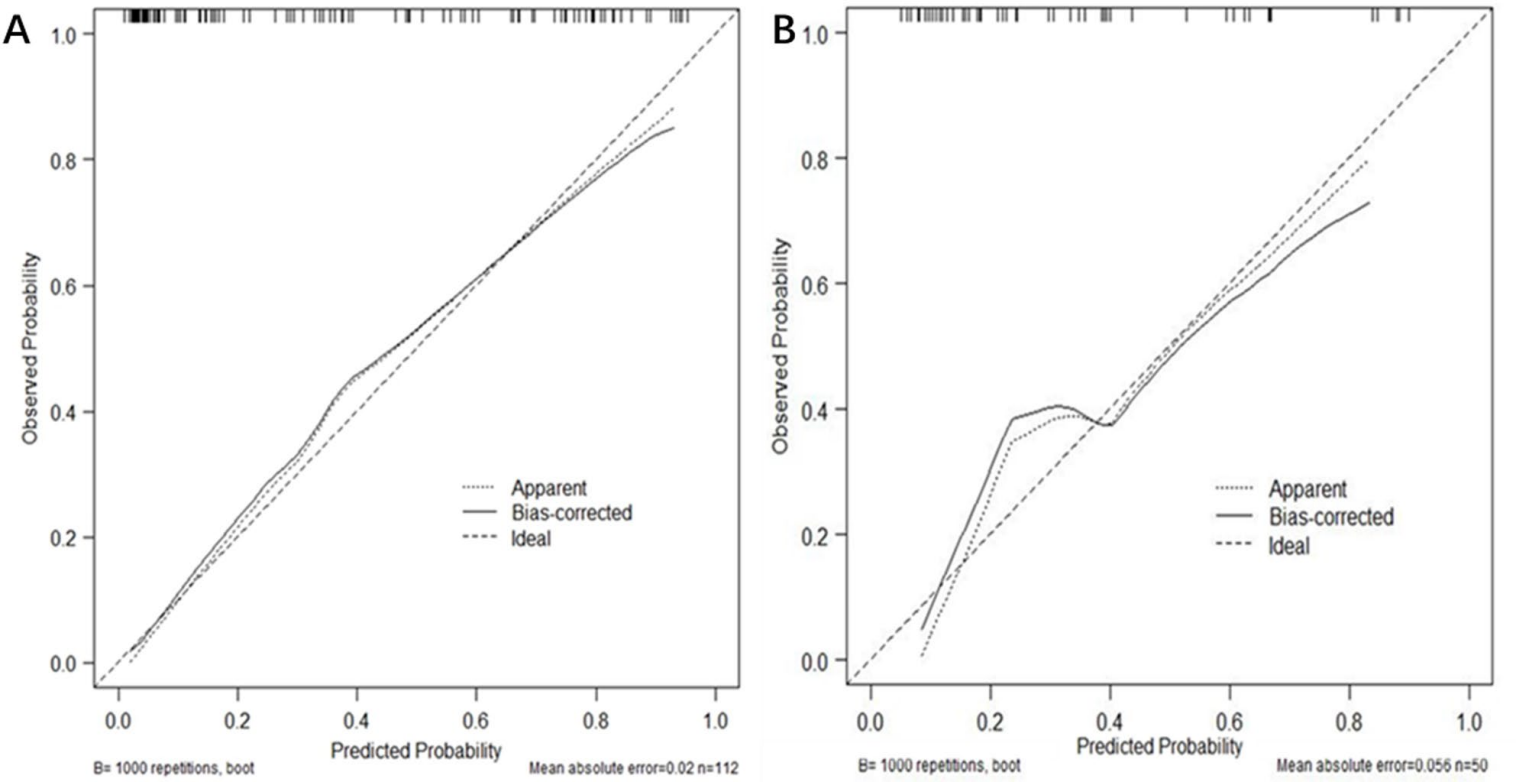
Calibration curves of the predictive model. **A** Calibration curve in the training cohort. **B** Calibration curve in the testing cohort

**Fig. 6:**
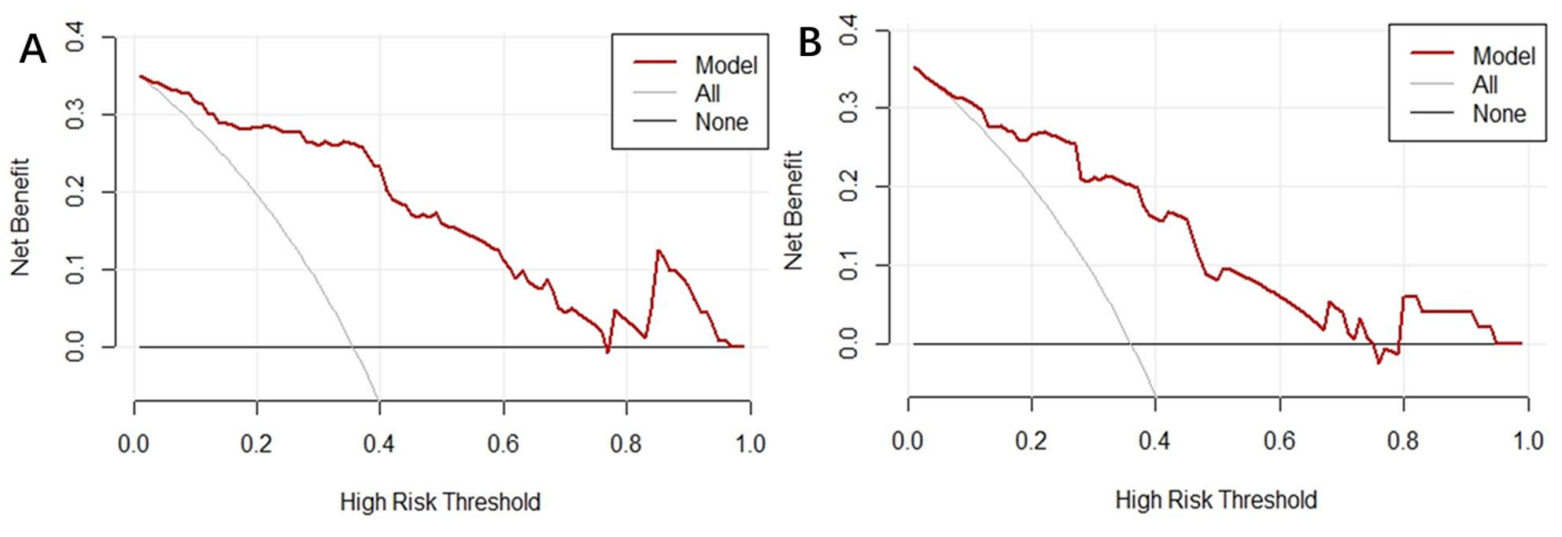
DCA of the predictive model. **A** DCA in the training cohort. **B** DCA in the testing cohort. Abbreviations: DCA=decision curve analysis

The nomogram based on these three CT features demonstrated good discriminative performance in both the training and testing cohorts, with AUCs of 0.901 (95% CI, 0.846–0.956) and 0.864 (95% CI, 0.737–0.937), respectively (Fig. 4). In the training and testing cohorts, the sensitivities for predicting VPI were 90% and 83%, with corresponding specificities of 83.3% and 84.4%. Calibration performance was acceptable, with low mean absolute error (MAE = 0.02 in the training cohort and 0.056 in the testing cohort), indicating limited deviation between predicted and observed risks and only a modest decrease in performance between the two cohorts. The calibration curves showed close agreement between predicted and observed VPI probabilities across the range of risk, and this agreement was preserved after bootstrap resampling (B = 1,000), as reflected by the bias-corrected curves (Fig. 5). Decision curve analysis suggested potential utility in preoperative risk assessment (Fig. 6).

## Discussion

Non-small cell lung cancer (NSCLC) remains a leading cause of cancer-related mortality worldwide, and lung adenocarcinoma (LA) is its most common subtype [22, 23]. Visceral pleural invasion (VPI) is a clinically relevant adverse feature that affects staging, treatment decisions, and prognosis in LA [21, 24–27]. Previous studies have reported that VPI occurs more frequently in solid nodules than in subsolid nodules, possibly reflecting their generally higher invasive potential and propensity to breach the pleural barrier [28, 29]. Importantly, solid nodules with pleural contact represent a specific anatomic setting characterized by direct tumor–pleura apposition with minimal intervening lung parenchyma, which may lower the effective barrier to pleural invasion; therefore, conclusions drawn from subsolid or mixed cohorts may not generalize. In this context, we developed and internally validated a CT-based nomogram using spiculation, pleural indentation, and pleural contact length to estimate VPI risk in solid pulmonary adenocarcinoma nodules with pleural contact. Recent randomized trials, including JCOG0802/WJOG4607L and CALGB/Alliance 140,503, have supported sublobar resection as an acceptable option for selected patients with small-sized peripheral NSCLC. Against this evolving surgical background, preoperative VPI risk estimation may provide supplementary information in pleura-abutting solid nodules, particularly when the adequacy of limited resection is under consideration. However, because the role of VPI in determining the extent of resection remains controversial, the proposed nomogram should be interpreted as a supplementary risk-estimation tool rather than a stand-alone determinant of surgical strategy. The incremental contribution of the present study lies primarily in its subgroup-specific clinical focus rather than methodological complexity. Specifically, we developed a CT-based nomogram for solid pulmonary adenocarcinoma nodules with pleural contact using routinely assessed semantic CT features, thereby providing a simple and clinically deployable model for this particular clinical setting.

Spiculation has been proposed to reflect asymmetric infiltrative tumor growth along adjacent bronchi, vessels, and lymphatic channels, accompanied by stromal proliferation and neovascularization; on CT, it typically appears as fine radiating or corona-like strands extending from the nodule margin [30, 31]. Clinically, the presence of spiculation is often associated with a more aggressive phenotype and poorer prognosis [32]. A prior meta-analysis further demonstrated a significant association between spiculation and VPI (OR = 2.581, 95% CI: 1.640–4.062), supporting spiculation as an important semantic CT feature for preoperative VPI risk stratification [33]. Chen et al. [19] reported that spiculation was associated with an increased risk of VPI in pleura-contacting lung adenocarcinoma (OR = 2.70), and that combining spiculation with a pleural contact length > 10 mm improved sensitivity to 90%, with an AUC of 0.73. In our cohort, spiculation was present in 42.0% of nodules overall and was substantially more frequent in the VPI-positive group (70.7%). Moreover, multivariable logistic regression identified spiculation as an independent predictor of VPI in solid pulmonary adenocarcinoma nodules with pleural contact, consistent with the observations reported by Chen et al. However, the independent contribution of spiculation to VPI prediction has not been consistently reported across studies. For instance, Wang et al. investigated CT features associated with VPI in subpleural clinical stage IA lung adenocarcinoma and found that spiculation was significantly more common in the VPI-positive group than in the VPI-negative group (47.5% vs. 12.1%); nevertheless, spiculation was not retained as an independent risk factor in their multivariable model [34]. Such discrepancies may be attributable to differences in study populations and tumor–pleura interfaces. Notably, our study specifically focused on solid nodules with pleural contact, whereas other cohorts may have included lesions without pleural contact or with only indirect pleural proximity. Taken together, we speculate that the predictive value of spiculation may be more pronounced in tumors with pleural contact, potentially because these lesions exhibit stronger local invasiveness and are more likely to breach the visceral pleural barrier; this may partly explain why spiculation emerged as an independent predictor in our cohort.

The pleural indentation sign is believed to arise from intratumoral fibrosis, scar formation, and connective-tissue remodeling that generate traction on adjacent structures. When this traction becomes sufficiently strong, it can pull the visceral pleura inward toward the lesion and deform the pleural surface. In many cases, the adjacent pleura shows no overt thickening or adhesion, suggesting a localized pulling effect rather than diffuse pleural disease. On CT, the sign appears as focal inward retraction or wrinkling of the visceral pleura next to the nodule [35]. Previous studies [14, 34, 36, 37] reported pleural indentation as an independent predictor of VPI, possibly because it reflects tumor–pleura interaction and early pleural involvement. In our cohort of solid pulmonary adenocarcinoma nodules with pleural contact, pleural indentation was present in 55.0% of VPI-positive cases versus 34.7% of VPI-negative cases. After adjustment in multivariable logistic regression, the between-group difference remained statistically significant, supporting pleural indentation as an independent predictor of VPI. This relationship may relate to the biology of predominantly solid tumors—higher cellularity, a limited lepidic (ground-glass) component, and faster proliferation—which could promote local extension toward the pleura and increase the likelihood of both pleural indentation and VPI.

In the multivariate logistic regression analysis, pleural contact length (OR = 1.247, 95% CI: 1.120–1.387, *P* < 0.001) was identified as an independent predictor of VPI in solid pulmonary adenocarcinoma nodules with pleural contact. Previous studies have also shown that pleural contact length is independently associated with VPI. Chen et al. [19] reported that a contact length > 10 mm (*P* = 0.001) served as an independent predictor of VPI in solid pulmonary adenocarcinoma nodules. Deng et al. [38] found that, in peripheral lung adenocarcinoma with pleural contact, both the total pleural contact length (15.4 mm vs. 12.2 mm, *P* = 0.001) and the solid-component contact length (11.7 mm vs. 4.2 mm, *P* < 0.001) were significantly greater in the VPI-positive group, and the solid-component contact length remained an independent risk factor for VPI (*P* < 0.001). These findings are broadly consistent with our results. However, our findings differ from those of Heidinger et al. [39], who found that although there was a difference in contact length between the VPI-positive and VPI-negative groups, it was not an independent predictor of VPI. This discrepancy may be attributed to differences in measurement methods: Heidinger et al. measured linear contact length, while our study measured curved contact length, which may more accurately reflect the extent of contact between the nodule and the pleura. Overall, although pleural contact length cannot replace biopsy, it serves as a non-invasive indicator suggesting the presence of VPI and plays a crucial role in staging diagnosis, treatment planning, and prognostic evaluation.

Previous studies have shown that lung adenocarcinoma nodules with VPI exhibit more aggressive imaging features, which is consistent with our findings [16, 40, 41]. In this study, tumors in VPI-positive solid pulmonary adenocarcinoma patients were larger [18.9 ± 4.7 mm vs. 14.5 ± 4.9 mm, *P* < 0.001], had longer pleural contact lengths [19.1 (14.4, 22.7) mm vs. 9.2 (5.6, 13.6) mm, *P* < 0.001], and had higher frequencies of spiculation (70% vs. 26.4%, *P* < 0.001) and pleural indentation (55% vs. 34.7%, *P* = 0.037). Previous studies have reported tumor size as an independent risk factor for predicting VPI [16]. However, in our multivariable logistic regression analysis, tumor size was not identified as an independent risk factor for VPI, which may be related to differences in case selection and measurement methods. These imaging features have been associated with higher malignancy potential and poorer prognosis [42–44].

In this study, the nomogram showed good predictive performance in both the training and testing cohorts, with AUCs of 0.901 (95% CI: 0.846–0.956) and 0.864 (95% CI: 0.737–0.937), respectively. Previously published VPI prediction models can be broadly categorized into CT semantic-feature-based models, radiomics-based models, and combined models. These studies differ not only in model performance, but also in cohort composition, model inputs, validation strategy, interpretability, and intended clinical use. For example, Huang et al. [37] developed a multicenter nomogram combining CT features and radiomics features for subpleural invasive adenocarcinoma ≤ 2 cm, whereas Wang et al. [45] and Zhao et al. [46] proposed radiomics-based or combined models in broader early-stage lung adenocarcinoma cohorts. By comparison, the present study specifically focused on solid pulmonary adenocarcinoma nodules with pleural contact, a clinically specific subgroup characterized by direct tumor–pleura apposition and potentially distinct VPI risk patterns. In addition, unlike radiomics-based approaches that rely on lesion segmentation and may be sensitive to image acquisition and reconstruction heterogeneity, our model was constructed using routinely assessed CT semantic features and pleural contact length, which may offer greater interpretability and facilitate implementation in routine preoperative assessment. However, we do not claim superiority over previously published models based solely on numerical performance, because no direct head-to-head comparison was performed within the same cohort. Rather than competing on technical complexity, we aimed to provide a pragmatic tool for supplementary preoperative VPI risk estimation in routine clinical assessment. In current thoracic surgical practice, the choice of operative procedure is determined by multiple factors, including tumor size, radiologic invasiveness, expected surgical margin, pulmonary reserve, and patient comorbidities. Therefore, the proposed nomogram is not intended to serve as a stand-alone determinant of treatment strategy. Rather, it may provide supplementary preoperative information in selected borderline clinical scenarios involving solid nodules with pleural contact. Importantly, because the present study did not evaluate the relationship between predicted VPI risk and actual surgical procedures or postoperative outcomes, its potential impact on clinical management remains to be established in future studies. Accordingly, the relative value of different VPI prediction models should be interpreted in light of their target populations and intended clinical settings. More complex radiomics-based models may provide additional quantitative information, whereas semantic-feature-based models such as ours may offer better interpretability and easier integration into routine workflow. Future studies with direct head-to-head comparison in external cohorts would help better define the complementary roles of these approaches.

This study has several limitations. It was a retrospective single-center study with a relatively limited sample size, which may introduce selection bias and restrict generalizability. Although the use of a single institutional dataset may provide relative consistency in CT acquisition, surgical workflow, and pathological assessment, it may also limit the transportability of the model to other institutions with different patient-selection pathways, imaging protocols, reconstruction parameters, and clinical practice patterns. The current 70%/30% split-sample approach constitutes internal validation rather than true external validation, and the relatively limited number of VPI-positive cases may affect model stability and restrict generalizability. Therefore, the present findings should be interpreted cautiously. In addition, because the current model was developed and internally validated within the same institutional setting, its broader applicability to external populations remains uncertain. We also did not perform subgroup analysis according to the anatomical location of pleural contact (e.g., costal, mediastinal, diaphragmatic, or fissural pleura), and such anatomic heterogeneity may influence the risk of VPI. Although the final model was kept parsimonious and no problematic multicollinearity was identified, the relatively limited number of VPI events warrants cautious interpretation of model stability. Further multicenter studies with external validation in independent cohorts are needed to further assess the reproducibility, robustness, and clinical applicability of the model across institutions with different patient populations, imaging protocols, and clinical workflows.

## Conclusion

In summary, we identified spiculation, pleural indentation, and pleural contact length as independent predictors of VPI in solid lung adenocarcinoma nodules with pleural contact and developed a CT-based nomogram for preoperative risk estimation. The model demonstrated good discrimination and acceptable calibration under internal validation conditions, and decision curve analysis suggested potential utility in preoperative risk assessment, thereby supporting its value as a supplementary tool for estimating VPI risk before surgery. Future multicenter studies with external validation are needed to further clarify its clinical integration, robustness, and broader applicability.

## Supplementary Information

Below is the link to the electronic supplementary material.

Supplementary Material 1

## Data Availability

The data underlying this article are not publicly available due to patient privacy and institutional restrictions, but will be shared on reasonable request to the corresponding author.

## Declarations

### Author information

Rui Xu- Master of Medicine candidate, Jianing Tang- Master of Medicine candidate, Qianyao Yuan- Master of Medicine candidate, Mengjiao Ding- Master of Medicine candidate, Jingjing Zhang- Master of Medicine candidate, Wenjun Yao-Doctor of Philosophy, Dai Zhang-Doctor of Philosophy, Hong Zhao -Doctor of Philosophy.

## Abbreviations

CT: Computed Tomography
VPI: Visceral Pleural Invasion
ROC: Receiver Operating Characteristic Curve
NSCLC: Non-Small Cell lung Cancer
OR: Odds Ratio
AUC: Area Under The Curve
DCA: Decision Curve Analysis
TNM: Tumor–Node–Metastasis
