# Supplementary material for: CT-based nomogram for estimating visceral pleural invasion in solid pulmonary adenocarcinoma nodules with pleural contact: a retrospective single-center study
Source: BMC Med Imaging. 2026 May 26;26:364. doi: 10.1186/s12880-026-02458-1 (PMC13390293; doi:10.1186/s12880-026-02458-1)
Supplement: Supplementary file 1 — Supplementary Material 1 [file 12880_2026_2458_MOESM1_ESM.docx]

Table 1 Inter-observer consistency analysis of CT features

| Qualitative indicators | Kappa value(95%CI) | Quantitative indicators | ICC(95%CI) |
| --- | --- | --- | --- |
| Nodule location | 1 | Nodule size | 0.991(0.987-0.994) |
| Spiculation | 0.908(0.830-0.986) | Pleural contact length | 0.994(0.992-0.996) |
| Lobulation | 0.854(0.756-0.952) |  |  |
| Air bronchogram | 0.936(0.865-1.000) |  |  |
| Vacuole sign | 0.951(0.884-1.018) |  |  |
| Pleural indention | 0.891(0.807-0.975) |  |  |
| Cavity | 1 |  |  |
| Calcification | 1 |  |  |
| Emphysema | 0.905(0.813-0.997) |  |  |
| Vessel convergence | 0.873(0.775-0.971) |  |  |

CI, Confidence interval; CT, computed tomography
